# Coronary Embolization of Valsalva Thrombus Following Transcatheter Aortic Valve Implantation

**DOI:** 10.1016/j.jaccas.2025.104191

**Published:** 2025-07-23

**Authors:** Yasuo Tsuru, Kenichi Ishizu, Masaomi Hayashi, Shinichi Shirai, Kenji Ando

**Affiliations:** Department of Cardiology, Kokura Memorial Hospital, Kitakyushu, Japan

**Keywords:** aortic valve, myocardial ischemia, thrombosis, valve replacement

## Abstract

This report describes definitive diagnosis of coronary embolization of Valsalva thrombus after transcatheter aortic valve implantation by using serial multimodality imaging. Valsalva thrombus has been reported not to be related to an increased risk of adverse events, but this case suggests the need for future studies on the characteristics of Valsalva thrombus with high embolic risk to determine the indications for prophylactic anticoagulation.

## Graphical Abstract

**Figure:**
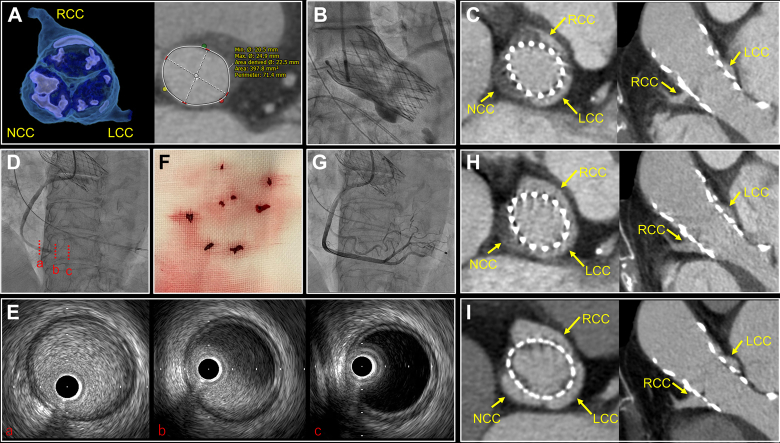

An 88-year-old woman with no comorbidity relevant to coagulation disorder was referred to our institution with symptomatic severe aortic stenosis. Computed tomography (CT) showed severely calcified leaflets with an annular perimeter of 71.4 mm (Figure 1A). Our heart team decided to perform transcatheter aortic valve implantation (TAVI) because of the high surgical risk. After predilatation with a 20-mm balloon, a 26-mm Evolut FX (Medtronic) was successfully implanted with trivial paravalvular leakage (Figure 1B). TAVI was performed under aspirin and intravenous heparin with an activated clotting time above 300 seconds throughout the procedure. Postprocedural cardiac CT, which is routinely performed the day after TAVI in our institution for assessing the leaflet thrombosis, commissural alignment, and feasibility of future redo TAVI, revealed complete thrombosis of the noncoronary cusp and partial thrombosis of the right coronary cusp (RCC) (Figure 1C). The commissural alignment was good. Because of no evidence of systemic embolization and valve dysfunction (mean aortic gradient: 5.8 mm Hg), oral anticoagulation was not initiated, and aspirin alone was continued.

**Figure 1:**
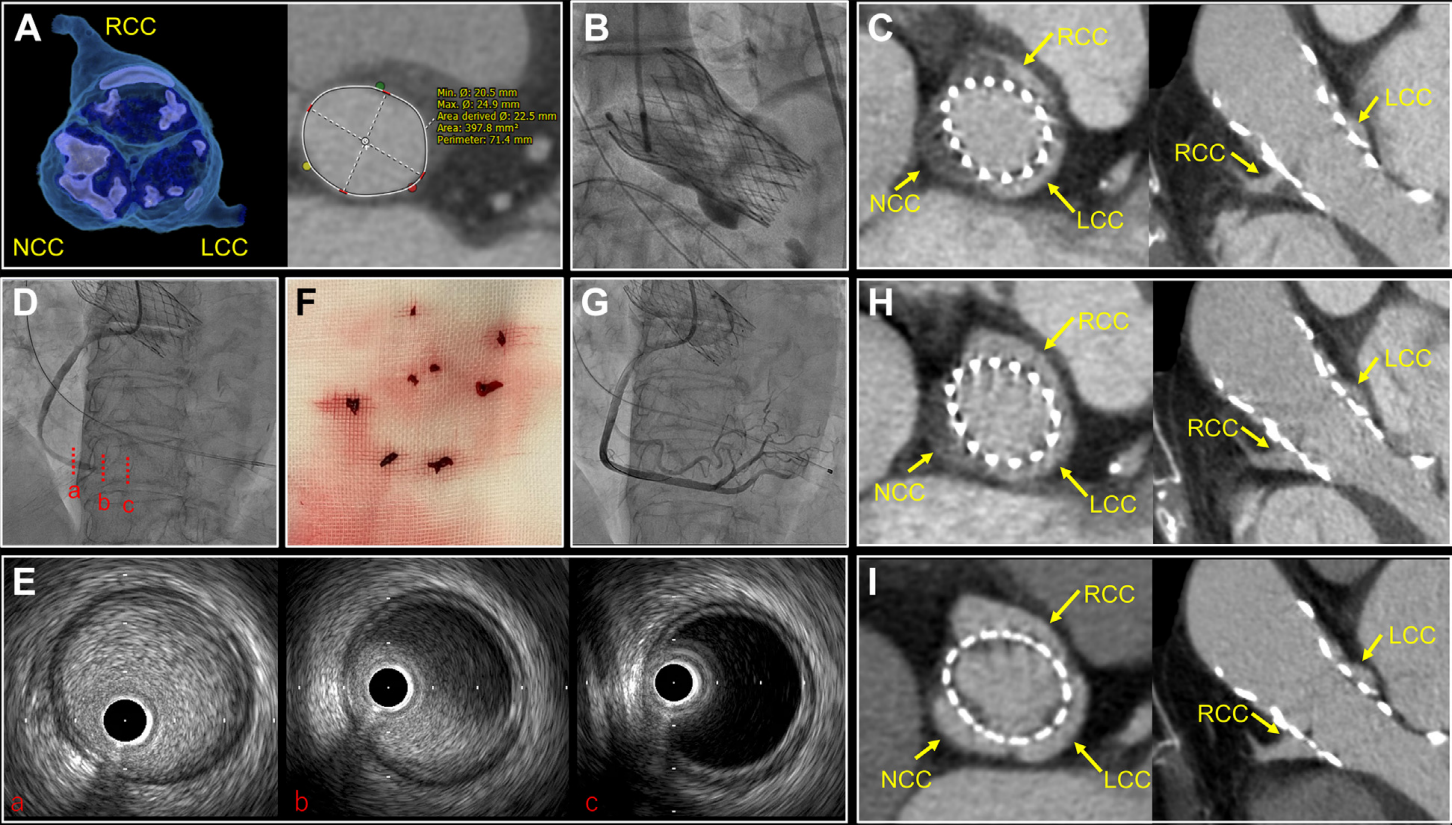
Coronary Embolization of Valsalva Thrombus Following TAVI (A) Preprocedural computed tomography (CT) images of aortic valve. (B) Aortography after 29-mm Evolut FX implantation. (C) CT images obtained the day after transcatheter aortic valve implantation (TAVI showing a thrombosis of the right coronary cusp and noncoronary cusp). (D) Emergent coronary angiography and (E) intravascular ultrasound showing thromboembolism of the right coronary artery. (F) Picture of aspirated thrombi. (G) Final coronary angiography showing successful revascularization. (H) CT images obtained immediately after percutaneous coronary intervention showing disappearance of right coronary cusp thrombus. (I) CT images 1 month following oral anticoagulation showing disappearance of noncoronary cusp thrombus. LCC = left coronary cusp; NCC = noncoronary cusp; RCC = right coronary cusp.

On day 2, the patient suddenly lost consciousness during rehabilitation. A 12-lead electrocardiogram revealed ST-segment elevation in leads II/III/aVF with complete atrioventricular block. Emergent coronary angiography and intravascular ultrasound revealed thromboembolism of the right coronary artery (RCA) (Figures 1D and 1E). Repeated thrombus aspiration resulted in successful revascularization (Figures 1F and 1G). CT obtained immediately after the coronary intervention confirmed the disappearance of the thrombus in the RCC (Figure 1H), suggesting that the thrombus had embolized to the RCA. One month after oral anticoagulation, CT revealed that the thrombus in the noncoronary cusp was also resolved (Figure 1I).

Valsalva thrombus has been reported in approximately 9% of patients after TAVI and is not related to an increased risk of adverse events.^1^ However, an RCC thrombus incidentally detected on post-TAVI CT embolized into the RCA in our case. As post-TAVI CT is not part of routine practice, future studies on the characteristics of Valsalva thrombus with high embolic risk, including baseline anatomic features and fluid dynamics of neosinus, are warranted to determine the indications for prophylactic anticoagulation.

## Funding Support and Author Disclosures

Dr Shirai is the proctor of transfemoral-TAVI for Edwards Lifesciences, Medtronic, and Abbott Medical. All other authors have reported that they have no relationships relevant to the contents of this paper to disclose.

**Take-Home Messages**

• Valsalva thrombus after transcatheter aortic valve implantation, which has reportedly not been associated with an increased risk of adverse events, caused embolization of the right coronary artery in our case.
• Anatomic features of Valsalva thrombus carrying higher embolic risk should be clarified in future studies to discuss the indications for preventive anticoagulation.
